# ﻿Penile shape discriminates two cryptic species of *Akodon* Meyen, 1833 (Mammalia, Rodentia, Cricetidae) from eastern Brazil

**DOI:** 10.3897/zookeys.1134.89587

**Published:** 2022-12-05

**Authors:** Leonardo Campana, Letícia Rosário Cruz, Roberta Paresque, Valéria Fagundes

**Affiliations:** 1 Departamento de Ciências Biológicas, Centro de Ciências Humanas e Naturais, Universidade Federal do Espírito Santo, 29.075-910, Vitória, Espírito Santo, Brazil; 2 Programa de Pós-graduação em Ciências Biológicas, Universidade Federal do Espírito Santo, 29.075-910, Vitória, Espírito Santo, Brazil; 3 Centro Universitário do Norte do Espírito Santo, Universidade Federal do Espírito Santo, 29.932-540, São Mateus, Espírito Santo, Brazil

**Keywords:** Glans penis, hybrids, interspecific variation, population variation

## Abstract

Glans penis morphology has been used as a powerful tool in mammal taxonomy to differentiate cryptic species. Neotropical rodent species *Akodoncursor* and *A.montensis* are cryptic, and interspecific hybrids are like their parental species. We investigated non-metric and metric phallic characters aiming to differentiate *A.cursor* from *A.montensis*. We also evaluated the parental species’ influence of the phallic characters on hybrids. We analysed 96 male adults—56 *A.cursor*, 27 *A.montensis*, and 13 hybrids, subgrouping species by locality and hybrids by parental species (paternal vs maternal). We verified that *A.cursor* and *A.montensis* are distinguishable by penile-shape morphology: *A.cursor* has an elongated penile form with a flare in the distal portion and *A.montensis* has a barrel-shaped form. Also, dark spots in ventral view, if present in *A.montensis*, distinguish *A.montensis* from *A.cursor*. Although the non-metric characters differentiate the species, they do not distinguish the subgroups of *A.cursor*, *A.montensis*, and hybrids. The metric phallic characters indicated a significant difference between species and hybrids. These characters also differentiate the population groups of *A.cursor*. However, *A.montensis* subgroups and hybrids subgroups did not present a significant difference. This study shows the importance of penis morphology in the taxonomy of the cryptic rodent species *A.cursor* and *A.montensis*, representing a powerful tool to discriminate male specimens in mammal collections without karyotyping or sequencing, even though the specimens occurred in sympatric areas. Since most taxidermy protocols do not preserve the penis in mammal preparations, liquid preservation of some specimens or the removal of the penis before taxidermy for liquid preservation could be beneficial. We also recommend the organisation in museum collections of a penis bank for the *A.cursor* species group (or even all rodent species) to avoid losing this important information for species identification.

## ﻿Introduction

*Akodon* Meyen, 1833 (Mammalia, Rodentia, Cricetidae) is the most diverse genus of the tribe Akodontini, with 42 species, one of the most speciose in the subfamily Sigmodontinae, and is widely distributed throughout South America (Pardiñas et al. 2015; Brandão et al. 2021, 2022). Among *Akodon* species, *A.cursor* (Winge, 1887) and *A.montensis* Thomas, 1913 are cryptic and sister species, being members of the *Akodoncursor* species group. *Akodoncursor* is endemic to eastern Brazil, distributed from the north in the states of Paraíba, Pernambuco, Alagoas and Bahia, throughout Minas Gerais, Espírito Santo, Rio de Janeiro and São Paulo, up to south in the state of Paraná. *Akodonmontensis*, in turn, occurs in Paraguay, northern Argentina, and in Brazil in states of Rio Grande do Sul, Santa Catarina, Paraná, Mato Grosso do Sul, São Paulo, Rio de Janeiro, and Minas Gerais, in the southeastern. These two species were found in sympatry in the states of Minas Gerais, Rio de Janeiro, São Paulo, and northern Paraná (Fagundes et al. 1998; Fagundes and Nogueira 2007; Valdez and D’Elía 2013; Pardiñas et al. 2015).

Despite being undistinguished by morphology, the karyotype information has been a diagnostic feature to identify these two species, as each species has very distinctive karyotypes based on the diploid number, the fundamental number (or number of autosome arms), and the polymorphisms of some autosome pairs. *Akodoncursor* has three diploid numbers (2n = 14, 15, and 16) and nine different fundamental numbers (FN = 18–26), which are due to a combination of pericentric inversions and centric fusions in five autosomal pairs (Sbalqueiro and Nascimento 1996; Fagundes et al. 1997; Fagundes et al. 1998). On the other hand, *A.montensis* presents a basic karyotype with 2n = 24 and FN = 42, with variation of diploid number due to the monosomy of X (2n = 23) or the presence of 1–3 supernumerary chromosomes (2n = 25–27) (Soares et al. 2018). Furthermore, each species has a very particular karyotype, with different pairs of autosomes involved in polymorphic rearrangements, making their identification by karyotype effective. Interspecific hybrids have already been reported, as wild-caught animals in forests of São Paulo or from offspring of crosses in captivity. They are easily distinguished from parentals by having cells with 2n = 19 or 20, due to fusions of the gametes of *A.montensis* (n = 12) with *A.cursor* (n = 7 or n = 8) (Fagundes et al. 1998).

Despite being easily characterised by karyotype, the search for strong characters to morphologically distinguish these two species has challenged researchers for decades. So far, these species differ from each other by the relative size of the upper molars, which are larger than 4.4 mm in *A.cursor* but smaller in *A.montensis* (Geise et al. 2005; Gonçalves et al. 2007); the absence of the gallbladder in *A.montensis* and its presence in *A.cursor* (Geise et al. 2004); the cranial size and shape (Astúa et al. 2015), and the microstructure of the hair (Silveira et al. 2013). The need of dissecting animals to verify the presence of a gallbladder and using a magnification to analyse other characters are some of the difficulties encountered in identifying these two species by morphology.

In the last decade, several authors have shown the effectiveness of external penile features to differentiate mammals’ species that are very close morphologically (e.g., Rocha-Barbosa et al. 2013; Cserkész et al. 2016, 2019; Comelis et al. 2018; Fasel et al. 2020). In the past, phallic apparatus morphology was of importance in the systematics of mammals, giving information on the interrelationship of species (Hooper 1958; Lidicker 1968; Balbontin et al. 1996). Thus, new perspectives for taxonomy pointed that penile features may be remarkable for rodents and may provide important characteristics to help distinguish cryptic species (Adebayo et al. 2011).

Therefore, this study aims to provide an additional tool to distinguish *A.cursor* and *A.montensis*. Looking for intra- and interspecific variations, we evaluated non-metric and metric penile characteristics of *A.cursor*, *A.montensis*, and their hybrids using an unprecedented sample including wild- and captive-born individuals. We also evaluated if parental species (paternal or maternal) influenced the composition of phallic forms in hybrids.

## ﻿Methods

### ﻿Sampling

**Figure 1.**
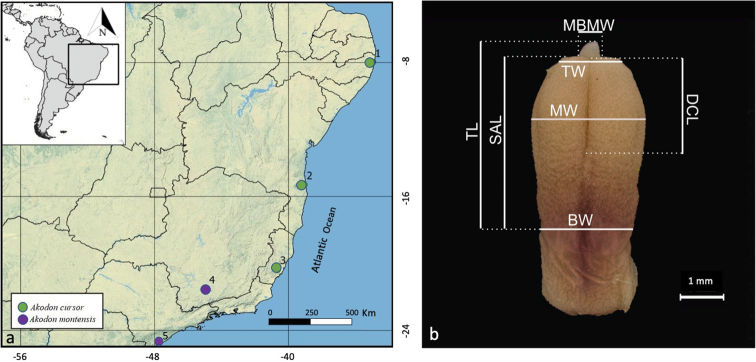
**A** sampling sites of wild-born specimens in Brazil: (1) Camaragibe, Pernambuco state (−8.02, −34.99); (2) Una, Bahia state (−15.30, −39.07); (3) Domingos Martins, Espírito Santo state (−20.37, −40.67); (4) Luminárias, Minas Gerais state (−21.57, −44.79), and (5) Ilha Comprida, São Paulo state (−24.73, −47.55) **B** glans penis of *A.cursor* in dorsal view. Abbreviation of measurements indicated in the image: spined area length (SAL), total length (TL), base width (BW), tip width (TW), middle width (MW), dorsal cleft length (DCL), and medial bacular mound width (MBMW).

For penis analyses, we used 96 male adults (Table 1, Fig. 1A, Suppl. material 1), including 14 wild-born (11 *A.cursor* and 3 *A.montensis*) and 82 captive-born (56 *A.cursor*, 27 *A.montensis*, and 13 hybrids). See Table 1 for subgroup acronyms. For captive-born grouping, we analysed individuals aged at least 3 months old. The species identification and their hybrids were based on karyotype features: diploid number (2n), the number of autosomal arms or fundamental number (FN), and the morphology of autosomes. Metaphases were obtained from bone marrow after *in vivo* injection of colchicine, following Fagundes et al. (1998). All wild-born individuals were deposited in the Mammalian Collection at Universidade Federal do Espírito Santo (UFES-MAM), Vitória, Brazil. The captive-born individuals were kept in the Mammalian Collection of the Laboratory of Animal Genetics in UFES. For euthanization, we used xylazine (Sedanew) and ketamine (Quetamina), following the recommendation of the Animal Ethics Committee from UFES (CEUA-UFES), under process 037/2015.

**Table 1.** Sampling of *Akodon* species and their hybrids, considering wild- and captive-born individuals, their origins, and hybrids, with the origin of parental species in experimental crossings.

Species	Subgroup acronyms	Wild-type individuals		Captive-born individuals		Total
		Origin	Number of individuals	Origin of parentals*	Number of individuals	
*A.cursor*	ACU^BA^	Una, BA	1	Una, BA × Una, BA	23	24
	ACU^ES^	Domingos Martins, ES	1	Domingos Martins, ES × Domingos Martins, ES	10	11
	ACU^PE^	Camaragibe, PE	9	Camaragibe, PE × Camaragibe, PE	12	21
*A.montensis*	AMO^SP^	Ilha Comprida, SP	3	Ilha Comprida, SP × Ilha Comprida, SP	13	16
	AMO^SP×MG**^	-	-	Ilha Comprida, SP × Luminárias, MG	11	11
Hybrids**	HYB^AMO×ACU^	-	-	Ilha Comprida, SP × Domingos Martins, ES	6	6
	HYB^ACU×AMO^	-	-	Una, BA × Ilha Comprida, SP	7	7
Total			14		82	96

BA = Bahia; SP = São Paulo; ES = Espírito Santo and MG = Minas Gerais states in Brazil. *For subgroups of captive-borns, the origin of paternal × maternal parentals, respectively. **The subscript represents the paternal × maternal parental species, respectively.

### ﻿Penile preparations and measurements

The penises were extracted using scissors, fixed in a 10% formalin solution for 24 h, and stored in 70% ethanol. Before analysis, they were air dried at room temperature and then photographed in ventral and dorsal views using an extended-focus imaging system (GT-Vision, Leica Microsystems). We used digital photographs with a scale bar to determine the linear measurements of each specimen.

Firstly, six non-metric characters of the penile morphology were described to identify the character states. We focused on the following characters: spine morphology, dorsal cleft, dorsal cleft depth, ventral cleft, glans shape, and presence/absence of at least one dark spot on the ventral side of the glans (Table 2).

**Table 2.** Non-Metric characters of the spines and glans penis with their respective character-states.

	Non-metric character	Character states
Spines (S)	Robust: base spine larger than 70 µm	0
	Intermediate: base spine with size >50 µm and <70 µm	1
	Narrow: base spine smaller than 50 µm	2
Dorsal cleft (DC)	Smaller: than half the length of the Spined Area	0
	Same size: as half the length of the Spined Area	1
	Larger: than half the length of the Spined Area	2
Dorsal cleft depth (DCD)	Shallow	0
	Intermediate between shallow and deep	1
	Deep	2
Ventral cleft (VC)	Absent	0
	Shallow	1
	Deep	2
Glans shape (GS)	Cylindric: long, cylinder shape with a flare distally	0
	Barrel-shaped: short, barrel shape without a flare distally	1
Dark spots on ventral view (DS)	Absent	0
	Present, with one or more spots	1

Then, using the TPSDig2 v. 2.31 (Rohlf 2009), we took seven linear measurements of the ventral and dorsal sides of the glans penises (Fig. 1B), as follows: total length (TL; the distance from the beginning of the dorsal base of the spined area to the distalmost point on the glans); spined area length (SAL; distance from the beginning of the dorsal base of the spined area to the distalmost point on the spined area); base width (BW; diameter of the glans at the beginning of the base of the spined area); meddle width (MW; greatest diameter of the glans penis, usually at the middle part of the spined area); tip width (TW; diameter of the distal end of the spined area); dorsal cleft width (DCL; distance from the distalmost point to the proximal-most point of the dorsal cleft); and medial bacular mound width (MBMW; diameter of the base of the medial bacular mound). To guarantee the reliability of the measuring methods, the measurements were taken five times by the same researcher.

We used scanning electron microscopy (SEM) in a subsample (one specimen of each morphology) to describe glans spines. SEM was performed at the Laboratory of Cellular Ultrastructure Carlos Alberto Redins at UFES (LUCCAR-UFES) using a JEOL JSM 6610 LV scanning electron microscope. Penises were dehydrated in three baths of 70%, 90%, and 100% ethanol for 30 min at each step, followed by two final baths in 100% ethanol. Samples were then dried by using a Autosamdri-815 automatic critical point dryer (Tousimis) and coated with gold using a desk V sample preparation system (Denton Vacuum).

### ﻿Statistical analyses

The six non-metric characters were analysed using PAST3 (Hammer et al. 2001). The similarity degree was assessed by classical clustering with 1000 replications (bootstrap) using Bray-Curtis distances to generate the current phenograms.

The seven metric characters were analysed using SPSS software statistics v. 26.0 (IBM 2019), and differences were considered significant when *p* < 0.05. To confirm the repeatability of the five repeated measurements for each character, we performed an ANOVA test. Then, all subsequent analyses were performed using the means of repeated measures for each specimen. The variables were also tested for normality with the Shapiro-Wilk and Kolmogorov-Smirnov test, and homoscedasticity with Levene’s test.

Once descriptive statistics were obtained for all groups and subgroups, we used an independent sample *t*-test to compare wild- and captive-born individuals of the same species (Pernambuco for *A.cursor* and from São Paulo for *A.montensis*), with our aim to find differences among each group.

To compare *A.cursor* (hereafter ACU), *A.montensis* (AMO), and hybrids (HYB), we arranged one general group of individuals per locality (Table 1). Hybrids were compared with their parental species. The mean values of statistical significance were estimated using a *t*-test for independent samples to compare only two groups.

For the comparative analyses of three groups, we performed a one-way ANOVA to evaluate which variables have significant differences among groups. Following the ANOVA, multiple comparison tests (Tukey’s HSD) established which group differed from one another.

We also performed a linear discriminant analysis for the metric characters on PAST3 to access the pattern of the morphological traits that differentiate the groups.

## ﻿Data resources

The data underpinning the analysis reported in this paper are deposited in the Global Biodiversity Information Facility (GBIF) and are available at https://doi.org/10.15468/24hz7g.

## ﻿Results

### ﻿Glans penis morphology

**Figure 2.**
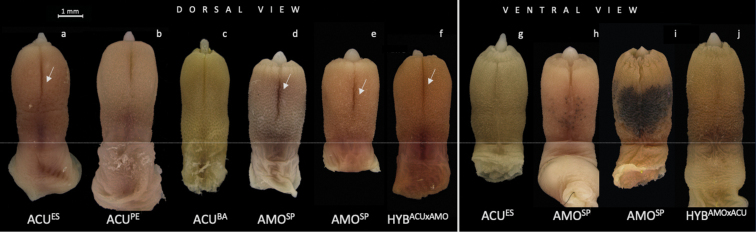
Representatives of the glans penis in dorsal and ventral views of *A.cursor* from Espírito Santo (ACU^ES^), Pernambuco (ACU^PE^), and Bahia (ACU^BA^) states; *A.montensis* from São Paulo state (AMO^SP^); hybrids from *A.cursor* paternal and *A.montensis* maternal parental (HYB^ACU×AMO^) and hybrids from *A.montensis* paternal and *A.cursor* maternal parental (HYB^AMO×ACU^). The horizontal line delimits the beginning of the glans spined area **A** elongated shape, dorsal cleft as long as half of the length of the spined area, with a deep dorsal cleft **B** elongated shape, dorsal cleft smaller than half of the length of the spined area, with intermediate dorsal cleft depth **C** elongated shape, dorsal cleft as long as half the length of the spined area, with a shallow dorsal cleft **D** barrel-shaped, dorsal cleft longer than half of the length of the spined area, with a deep dorsal cleft (at arrow) **E** barrel-shaped, dorsal cleft longer than half of the length of the spined area, with a deep dorsal cleft (at arrow) **F** barrel-shaped, dorsal cleft longer than half of the length of the spined area, with deep dorsal cleft (at arrow) **G** elongated shape with an absent ventral cleft **H** barrel-shaped, with a deep ventral cleft and presence of small dark spots **I** barrel-shaped, with deep ventral cleft and presence of a large dark spot **J** elongated shape, with ventral cleft absent. Arrow points to the deep dorsal cleft. Scale bar: 1 mm.

**Figure 3.**
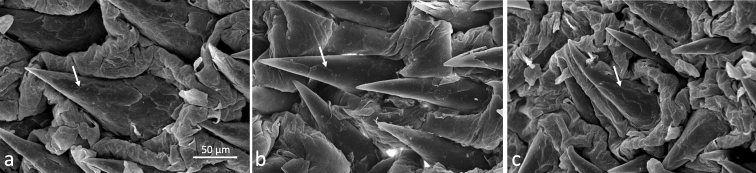
Electron microscopy (450×) of the spines on the dorsal side of the glans penis showing the three morphologies (at arrows) **A** robust **B** intermediate **C** narrow.

The medial bacular mound projects beyond the glans tip of the penis, and the glans surface is extensively covered by spines, which are thicker at the penile base and sharper at the tip (Fig. 2). Spines vary in size gradually along the glans, being larger at the base and narrower at the tip. All spines face toward the penile base and have three morphologies: (1) robust, with a base larger than 70.0 µm; (2) narrow, with the base less than 50.0 µm; and (3) intermediate, with the base between 50.0 µm and 70.0 µm (Fig. 3, Table 2). We detected two shapes of the glans penis: (1) elongated, with the distal part flared and larger in diameter than the proximal part; and (2) barrel-like in shape, with the central area more enlarged than the base and the tip. We also observed two clefts in the glans: a dorsal cleft which varied in length and depth among individuals, and a ventral cleft, which is always smaller than the dorsal cleft.

### ﻿Comparison wild- and captive-born individuals

Firstly, we tested whether wild- and captive-born individuals show significant differences in their metric characters. The captive-born ACU^PE^ individuals (*n* = 12) showed no significant differences in metric characters compared to the wild-born ones (*n* = 9), likewise, the captive-born AMO^SP^ individuals (*n* = 13) showed no significant differences when compared to the wild-born ones (*n* = 3) (Suppl. material 2). Based on these analyses, we grouped captive- and wild-born individuals and set a total sample size of 19 ACU^PE^ and 16 AMO^SP^ individuals. The ACU^BA^ and ACU^ES^ populations consisted of only one wild-born specimen each, and a comparative analysis between wild- and captive-born individuals was not performed. Finally, for estimating interspecific and intraspecific variations, we used the total sample for each population, as indicated in Table 1.

#### Intraspecific variation in *Akodoncursor*

**Figure 4.**
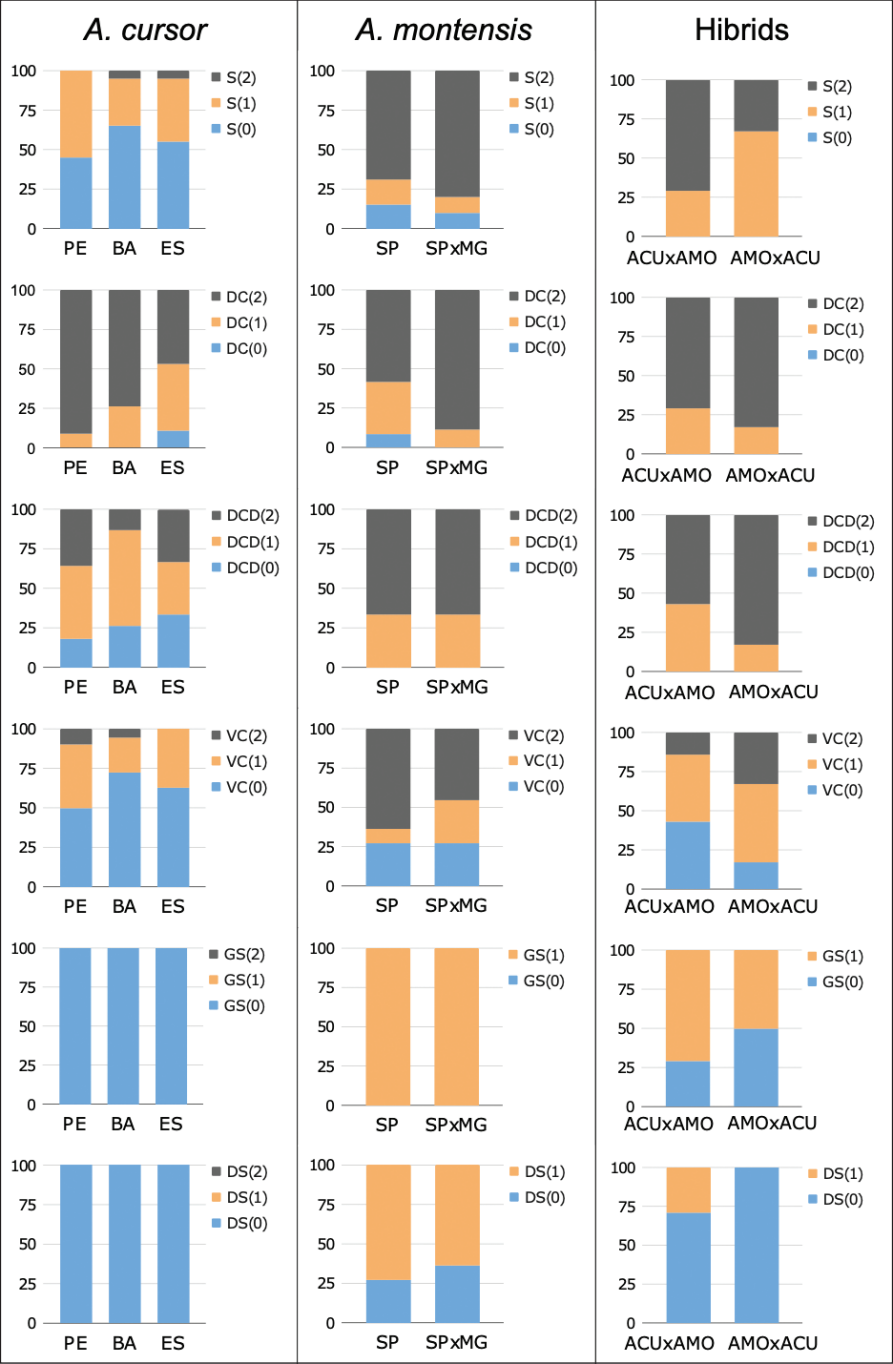
Frequency of the non-metric characters in the subgroups *A.cursor* from Pernambuco (PE), Bahia (BA), and Espírito Santo (ES) states; *A.montensis* from São Paulo (SP) and captive-borns from crossings between individuals from São Paulo and Minas Gerais states (SP×MG); and hybrids with paternal *A.cursor* and maternal *A.montensis* parentals (ACU×AMO), and with paternal *A.montensis* and maternal *A.cursor* parentals (AMO×ACU). Character abbreviation: S = spines; DC = dorsal cleft; DCD = dorsal cleft depth; VC = ventral cleft; GS = glans shape; DS = dark spots.

Based on our analysis of non-metric characters, all individuals of the ACU species presented a glans with an elongated morphology (Fig. 4, Suppl. material 3). All three spine morphologies (Fig. 3) were found among ACU^BA^, ACU^ES^, and ACU^PE^ subgroups, although the “robust” form was the most frequent for the ACU^BA^ (65.2%) and ACU^ES^ (55.0%) subgroups, while the “intermediate” form was more frequent for ACU^PE^ (45.4%).

Two morphologies of the dorsal cleft (“larger” and “same size” than half of the spined area) were observed on all ACU populations, while the dorsal cleft “smaller” than half of the spined area occurred only in ACU^ES^ population. The dorsal cleft “larger” than half of the spined area was the character with highest frequency among the three subgroups: ACU^BA^ (73.9%), ACU^PE^ (90.9%), and ACU^ES^ (47.3%).

The dorsal cleft presented three morphologies in ACU populations, with the “intermediate” morphology most frequent in ACU^BA^ (60.8%) and ACU^PE^ (45.4%), while for ACU^ES^ the three morphologies showed non-significant differences.

**Figure 5.**
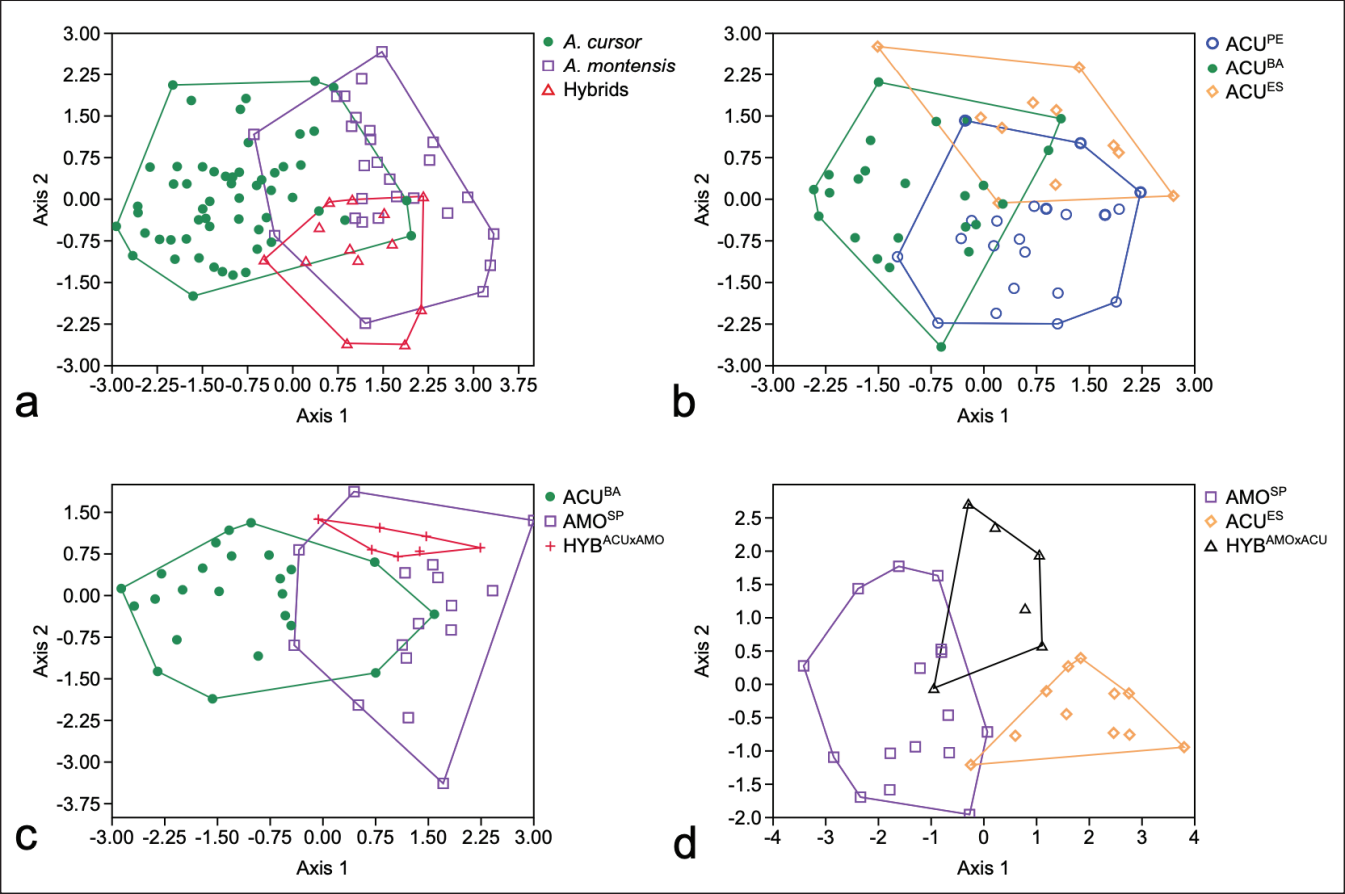
Linear discriminant analyses of **a***A.cursor* (green dot), *A.montensis* (purple square), and hybrids (red triangle) **b** locality groups of *A.cursor*: ACU^PE^ (blue circle), ACU^BA^ (green dot), and ACU^ES^ (orange diamond) **c** hybrid subgroup HYB^ACU×AMO^ (red cross) and their parental subgroups ACU^BA^ (green dot) and AMO^SP^ (purple square) **d** hybrid subgroup HYB^AMO×ACU^ (black triangle) and their parental subgroups AMO^SP^ (purple square) and ACU^ES^ (orange diamond).

All ACU populations showed the ventral cleft “absent” and “shallow” morphologies, with “absent” the most frequent: ACU^BA^ with 72.2%, ACU^PE^ with 50.0%, and ACU^ES^ with 62.5%. A “deep” ventral cleft was observed exclusively in ACU^ES^. Comparing the ACU subgroups, the discriminant function 1 explained 58.55% of the total variance. Overall, subgroups discriminant success was 76.79% (Fig. 5B). ACU^ES^ showed higher means for all variables (Table 3).

**Table 3.** Mean and *SD* of glans penis spined area length (SAL); total length (TL); base width (BW); tip width (TW); meddle width (MW); dorsal cleft length (DCL); medial bacular mound width (MBMW) of *A.cursor*, *A.montensis* and subgroups, and hybrids.

Group	N	SAL	TL	BW	TW	MW	DCL	MBMW
*A.cursor*	56	5.01 (±0.35)	5.76 (±0.38)	2.67 (±0.26)	1.89 (±0.28)	3.07 (±0.33)	2.63 (±0.32)	0.65 (±0.11)
*A.montensis*	27	4.73 (±0.35)	5.27 (±0.27)	2.78 (±0.22)	2.02 (±0.38)	3.17 (±0.30)	2.86 (±0.47)	0.70 (±0.15)
Hybrids	13	4.84 (±0.23)	5.58 (±0.23)	2.93 (±0.23)	1.95 (±0.24)	3.23 (±0.21)	3.01 (±0.25)	0.66 (±0.11)
ACU^BA^	24	5.03 (±0.29)	5.78 (±0.38)	2.68 (±0.22)	1.82 (±0.26)	2.98 (±0.27)	2.64 (±0.26)	0.61 (±0.10)
ACU^ES^	11	5.29 (±0.29)	6.06 (±0.43)	2.84 (±0.13)	2.00 (±0.24)	3.40 (±0.19)	2.88 (±0.21)	0.76 (±0.05)
ACU^PE^	21	4.85 (±0.36)	5.58 (±0.25)	2.56 (±0.24)	1.91 (±0.32)	3.01 (±0.35)	2.45 (±0.35)	0.63 (±0.10)
AMO^SP^	16	4.82 (±0.34)	5.28 (±0.27)	2.08 (±0.21)	1.98 (±0.35)	3.16 (±0.30)	2.73 (±0.50)	0.61 (±0.14)
AMO^SP-MG^	11	4.58 (±0.33)	5.26 (±0.27)	2.74 (±0.24)	2.08 (±0.44)	3.19 (±0.30)	3.04 (±0.38)	0.71 (±0.15)
HYB^ACU×AMO^	7	4.87 (±0.28)	5.47 (±0.24)	2.89 (±0.23)	1.84 (±0.21)	3.16 (±0.23)	2.99 (±0.22)	0.64 (±0.05)
HYB^AMO×ACU^	6	4.79 (±0.17)	5.70 (±0.15)	2.97 (±0.26)	2.09 (±0.21)	3.32 (±0.17)	3.04 (±0.30)	0.78 (±0.12)

Six out of seven metric characters showed significant differences in the one-way ANOVA test among the three subgroups of *A.cursor*: length of the spined area (SAL, *F*_2_ = 6.884; *P* = 0.002), total length (TL, *F*_2_ = 7.182; *P* = 0.002), width of the base (BW, *F*_2_ = 4.907; *P* = 0.011), width of the glans middle part (MW, *F*_2_ = 8.812; *P* < 0.001), length of the dorsal cleft (DCL, *F*_2_ = 8.030; *P* = 0.001), and width of the medial bacular mound (MBMW, *F*_2_ = 10.197; *P* < 0.001). Pairwise Tukey’s tests showed that ACU^ES^ showed a significant difference when compared with ACU^PE^ for all the ANOVA significant characters. Compared to ACU^BA^, ACU^ES^ showed a significant difference just for the characters middle width (MW, *P* = 0.001) and medial bacular mound width (MBMW, *P* = 0.000). On the other hand, ACU^BA^ did not present a significant difference when compared to ACU^PE^.

In short, *A.cursor* is mainly characterised by an elongated glans penis morphology, and no dark spots on the glans ventral side. The non-metric characters do not distinguish the subgroups in *A.cursor*. On the other hand, the metric characters were able to distinguish subgroup ACU^PE^ from ACU^ES^, while only two characters differentiated ACU^ES^ from ACU^BA^.

#### Intraspecific variation in *Akodonmontensis*

The two *A.montensis* subgroups were not distinguished by non-metric characters (Fig. 4, Suppl. material 3). All individuals showed glans with a barrel-shape form and the three spine morphologies, although the narrow morphology was the most frequent. The SP population presented the three dorsal cleft (DC) morphologies, and the DC larger than half of the spined area was the most frequent morphology in both subgroups. Black spots on the ventral side of the glans were exclusive to *A.montensis*; they were present in 64% and 73% of the individuals of the AMO^SP×MG^ and AMO^SP^ subgroups, respectively. When present, spots were either small and numerous (Fig. 2H) or single, large, and covering the three-quarters of the ventral side of the glans (Fig. 2I). Based on the metric characters, no significant differences were found between these subgroups using the independent samples *t*-test. In short, *A.montensis* can be characterised by a barrel-shaped glans penis and, if present, by dark spots in the ventral side.

#### Comparing interspecific variation between *Akodoncursor* and *A.montensis*

*Akodoncursor* and *A.montensis* were distinguished by the matrix of the six non-metric characters (Fig. 5A). Comparing the metric characters of each species, the discriminant analysis classified 79.2% of the two groups correctly, and the functions 1 explained 88.7% of the variance. In *A.cursor* the mean of the total length of the spined area was higher than in *A.montensis*. In *A.montensis* the means of the base width and dorsal cleft length were higher than in *A.cursor* (Table 3).

The *t*-test showed that *A.cursor* and *A.montensis* differed significantly by four characters: total length of the glans penis (TL, *t*_81_ = 5.917; *P* < 0.001), base width of the glans (BW, *t*_81_ = −2.006; *P* = 0.048), length of the spined area (SAL, *t*_81_ = 3.498; *P* = 0.001), and length of the dorsal cleft (DCL, *t*_69_ = −2.404; *P* = 0.019). While *A.cursor* (elongated glans penis) presented higher means for TL and SAL, *A.montensis* (barrel-shaped glans penis) presented higher BW and DCL.

**Figure 6.**
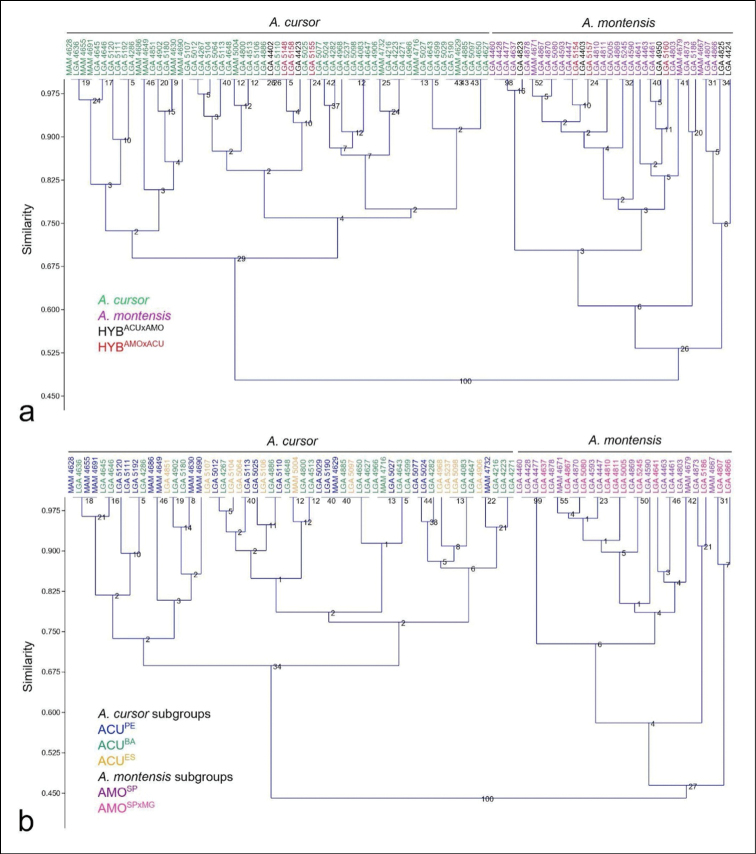
Classical clustering phenogram showing distinction of species group *A.cursor* and *A.montensis*. Bootstrap with 1000 replications **A** hybrids are arbitrarily distributed over groups, despite the origin of paternal and maternal parentals: *A.cursor* (green), *A.montensis* (purple), and HYB^ACU×AMO^ (black) and HYB^AMO×ACU^ (red) **B***A.cursor* and *A.montensis* subgroup representatives are also arbitrarily distributed over group species. Locality groups of *A.cursor*: ACU^PE^ (blue), ACU^BA^ (green), and ACU^ES^ (orange): *A.montensis* subgroups: AMO^SP^ (purple) and AMO^SP×MG^ (pink).

In short, seven non-metric characters differentiated *A.cursor* and *A.montensis* by classical clustering (Fig. 6). On the other hand, hybrids were not differentiated by non-metric characters. Of the metric characters, total length of the glans penis, base width of the glans, length of the spined area, and length of the dorsal cleft also differentiated *A.cursor* and *A.montensis*.

### ﻿Hybrids × parental variations

Although the matrix of the six non-metric characters found parental groups of *A.cursor* and *A.montensis* as separate, the hybrids were not separately clustered and showed no clear correspondence to either the paternal or maternal species group (Fig. 5A, C, D).

On the other hand, hybrids showed distinct frequencies of non-metric characters, depending on the species of the paternal and maternal parental species. With regards to the glans shape (GS), in the HYB^ACU×AMO^ subgroup (*n* = 7), in which ACU^BA^ is the paternal and AMO^SP^ is the maternal parental, 71% of the individuals had a barrel-shaped glans, like AMO species. In the HYB^AMO×ACU^ subgroup (*n* = 6), in which AMO^SP^ is the paternal and ACU^ES^ is the maternal parental species, we verified an even frequency of the two GS morphologies (Fig. 4, Suppl. material 3). The narrow spines were more frequent (71.0%) in the HYB^ACU×AMO^ subgroup, like the maternal AMO species; however, the HYB^AMO×ACU^ subgroup showed a higher frequency of intermediate spines (67.0%), and a lack of the robust spines, distinct from the maternal ACU^ES^. No subgroup had the DC smaller than half of the spined area. However, both subgroups showed a higher frequency of a larger dorsal cleft: HYB^ACU×AMO^ at 71.0%, and HYB^AMO×ACU^ at 83.0%. The shallow dorsal cleft was not present in either subgroup, but the deep dorsal cleft showed a higher frequency for HYB^ACU×AMO^ at 57.0% and HYB^AMO×ACU^ at 83.0%. The three ventral cleft morphologies were present on the two subgroups. For HYB^ACU×AMO^, the absent ventral cleft and the shallow ventral cleft showed the same frequency at 43.0%. Whereas HYB^AMO×ACU^ showed a higher frequency for the intermediate ventral cleft at 50.0%. Finally, only the HYB^ACU×AMO^ subgroup had dark spots on the ventral surface of the glans, at frequency of 69.0%.

Regarding metric characters, while the means of the spined area length and total length in *A.cursor* were higher than in *A.montensis*, the hybrids showed intermediate values. The mean values of the base width and the dorsal cleft length in hybrids were higher than in both parental species (Table 3). The one-way ANOVA test, followed by the pairwise Tukey’s test, comparing the hybrids and the parental species groups showed that the former presents a significant difference for three characters: the base width of the glans with a significant difference between *A.cursor* and hybrids (BW, *P* = 0.003), the length of the dorsal cleft between *A.cursor* and hybrids (DCL, *P* = 0.003), and the total length between *A.montensis* and hybrids (TL, *P* = 0.025). The independent samples *t*-test of the two hybrids subgroups showed significant differences only for the width of medial bacular mound (MBMW, t_11_ = −2.701; *P* = 0.016). The subgroup HYB^AMO×ACU^, which has *A.montensis* as the paternal parental species, showed the mean width of the medial bacular mound was higher than in HYB^ACU×AMO^, which has *A.cursor* as the paternal species (Table 3).

In comparing the subgroup HYB^ACU×AMO^ with its paternal ACU^BA^ and maternal AMO^SP^ subgroups, the discriminant function 1 explained 87.74% of the variance (Fig. 5C), with 80.85% of the specimens correctly classified. The only variable that showed significant difference in the one-way ANOVA was the total length (TL, F_2_ = 11.756; *P* < 0.001).

Comparing the subgroup HYB^AMO×ACU^ with its paternal AMO^SP^ and maternal group ACU^ES^ subgroups, the discriminant function 1 explained 82.27% of the variance (Fig. 5d), with 87.88% of the individuals correctly classified. One-way ANOVA showed significant differences for three of the seven metric characters: length of the spined area (SAL, F_2_ = 8.982; *P* = 0.001), total length (TL, F_2_ = 19.381; *P* < 0.001), and the width of the medial bacular mound (MBMW, F_2_ = 7.422; *P* = 0.002). Pairwise Tukey’s tests showed that for the length of the spined area, the only significant difference is between HYB^AMO×ACU^ and ACU^ES^ (SAL, *P* = 0.008). Hybrids showed a mean less than the ACU^ES^ mean for the length of the spined area. For the other two characters, total length (TL, *P* = 0.028) and width of the medial bacular mound (MBMW, *P* = 0.012), a significant difference was observed between HYB^AMO×ACU^ and AMO^SP^. In this case, hybrids showed mean values greater than *A.montensis* for total length and medial bacular mound.

## ﻿Discussion

In the *Akodoncursor* group, *A.cursor* and *A.montensis* show exceptional morphological similarity, despite karyological and molecular divergences, and the difficulties with field identification may hamper ecological and conservation research. Museum specimens without known diploid numbers or molecular data may be randomly clustered and generate inaccurate information on occurrence and geographic distributions of these species.

A previous study has described the phallic morphology of the akodont group, focusing on internal characters and in the morphology of the baculum (Hooper and Musser 1964), and pointed to a combination of characters that distinguished *A.montensis* from some other akodonts, including *A.cursor*. In their study, Hooper and Musser (1964) included only two individuals, which were then classified as *A.arviculoidescursor* (from Rio de Janeiro, Brazil) and *A.a.montensis* (from Misiones, Argentina). However, a more recent study by Pardiñas et al. (2015) considered the geographic distribution of the supposed species, and the sample from Argentina was thought to be representative of *A.montensis*, but that from Rio de Janeiro could represent either *A.cursor* or *A.montensis*. Pardiñas et al. (2015) mentioned internal differences for the Brazilian individual against the Argentine one: baculum slightly larger, lateral bacular mounds larger, and spinous dorsally, and the urethral flap longer. However, they wrote nothing about the glans shape.

To the best of our knowledge, this is the first study focusing on penile morphology as a diagnostic character for distinguishing between *A.cursor* and *A.montensis*. Herein, we were able to discriminate the cryptic species based solely on morphological characters of the glans penis: elongated with a distal flare in *A.cursor* and barrel-shaped in *A.montensis*. Furthermore, dark spots on ventral view, when present, were exclusive to *A.montensis*. From these findings, we propose a dichotomous diagnosis to differentiate these two cryptic species based on their glans penis morphology.

Considering that the present study included 2–4-month-old individuals only, we assume that the dark spots on the glans are not correlated to the old age of the individuals. However, this character could still be associated with populational variation because our São Paulo sample is formed by individuals and descendants from Ilha Comprida (São Paulo), and our analyses did not include individuals from southern Brazil or Argentina. Chromatic anomalies like piebaldism, leucism, albinism, and melanism has been described for very few rodent species in Brazil (Silva et al. 2020), and anomalous colours on some body parts may occur due to an excess or deficit of the production of melanin (Abreu et al. 2013). Therefore, dark spots on the glans penis are entirely new for this taxon.

In this work, we verified that all specimens had the glans penis completely covered by spines with a thick base and a sharpened tip. Although we have observed three spine morphologies among *A.cursor* and *A.montensis*, at frequencies specific to each species, *A.cursor* showed a higher frequency of robust spines while *A.montensis* had higher frequency of narrow spines. However, the spine morphology was insufficient to differentiate the two species.

According to Rocha-Barbosa et al. (2013), complex penises and ornamentations, like spines, can have several functions, such as inducing ovulation through vaginal stimulation. This stimulation is caused by the friction of the penile spines on the vaginal wall. Solitary rodent species with seasonal breeding and a promiscuous mating system generally present induced ovulation, and the males are generally more often have penile ornamentation (Parag 2006).

Promiscuous mating is typical for both *A.cursor* and *A.montensis*, and this equates to having complex penises and the presence of spines (Gentile et al. 1997; Rosalino et al. 2013). The sperm competition in promiscuous mating systems may also be influenced by spines on the glans, which have the function of removing sperm present on vagina from previous mattings (Parag et al. 2006). Spines can also be genital locks during the copulation by holding the walls of the vagina (Altuna and Lessa 1985). Therefore, describing and characterising these structures in glans penis in cryptic and sympatric species may help improve our knowledge about the still poorly studied reproductive system of genus *Akodon*, enabling researchers to initiate innovative studies on sperm competition and postcopulatory selective pressures driving the evolution of sperm morphology.

Four metric characters showed a significant difference between *A.cursor* and *A.montensis* (spined area length, total length, base width, and dorsal cleft length), and in general, the metrics were greater in *A.cursor* than in *A.montensis*. These results are in accordance with craniodental metric characters in which *A.cursor* is larger than *A.montensis* (Geise et al. 2005; Astúa et al. 2015). On the other hand, two other metrics not associated with size of the penis (base width and dorsal cleft length) were smaller in *A.cursor* than in *A.montensis*.

The *A.cursor* subgroups organised by locality were not separated in distinct clusters using non-metric characters. Qualitatively, *A.cursor* subgroups showed the same frequency of characters. However, all the metric characters showed differences between groups with the mean values of the Espírito Santo group (in the south) being significantly different than those of the Pernambuco and Bahia groups (in the north). The differentiation among Espírito Santo and Bahia/Pernambuco was also observed in cytochrome-b molecular data (Colombi et al. 2010; Maestri et al. 2016), which found a genetic differences between samples from south of the Jequitinhonha River and those of north of the river (including Bahia and Pernambuco). Even though the three groups are genetically distinct, individuals from Bahia to Pernambuco are morphologically similar. The variation in the penile metrics in distinct geographic groups can be explained by the already known variation in geographic groups of mammals. Even though we analysed three populations of *A.cursor*, we assume additional specimens using this approach would show new patterns to understand the overall population variability of this species.

One of the most outstanding results of this study is the data on *A.cursor* and *A.montensis* hybrids, in which qualitative characters are insufficient to differentiate hybrids from the parental species. The hybrids did not display an intermediate state for the glans shape, but instead displayed either one morphology or the other, like the parental species. It is noteworthy that most of the seven hybrid descendants had a barrel-shaped glans morphology (likewise *A.montensis*) when the maternal species was *A.montensis*. However, the other way around, when *A.cursor* was the maternal species, this predominance was not observed, and the glans shape varied equally between elongated and barrel-shaped. One could say that our findings should be cautiously considered, because of the small sample size of hybrids. We agree.

For the quantitative characters, the glans of the hybrid subgroup morphologies was not clearly associated with one of the parental species and the hybrids seem to have a distinct identity from their parental species, probably due to the combination of the expression of these characters independently. This pattern is observed in characters of the skull of subterranean tuco-tucos, whose combination of parental features generates a distinct configuration (Kubiak et al. 2020). Characters that display intermediate metrics compared to parentals seem to follow a complex multigenic determinism due to additive genetic variation in hybrids (Renaud et al. 2012). On the other hand, we can observe some examples of hybrids that follow one of the parental species. Hybrids between *Musmusculusdomesticus* and *M.musculus* showed a skull-shape gradient more like one parent than the other one (Auffray et al. 1996). Bat hybrids *Myotismyotis* and *M.oxygnathus* showed mixed results in skull traits, with the skull size closer to one parental species but the tooth row construction closer to the other species (Bachanek and Postawa 2010).

Our study showed the importance of penis morphology in the taxonomy of the rodent cryptic species *A.cursor* and *A.montensis*. Our results represent a powerful tool that allows us to identify male specimens in fieldwork, without karyotyping or sequencing, especially in sympatric areas. On the other hand, the absence of an intermediate or a unique shape for hybrids could result in an imprecise identification for this group in sympatric areas. Therefore, we strongly recommend the concatenate analysis of the morphology of the dorsal spines with the glans penises. Only individuals of *A.cursor* presented the combination of elongated glans and robust spines, a profile that can help differentiate this species from *A.montensis* and their hybrids when in sympatry.

Our work contributes by bringing new strategies to facilitate specimen identification in the field with the naked eye or with the help of a magnifying glass. However, considering that the observation of the penile glans in animals that are awake can be very stressful, analyses of the penile glans should minimally be performed in anaesthetised individuals. For identification of specimens in museum collections, on the other hand, we strongly recommend that the penile is important and must be preserved. Since most taxidermy protocols do not include the penis in mammal preparations, in our interpretation, we consider as essential that some individuals be preserved whole in ethanol or, if not possible, at least the penis be removed and preserved. We recommend that there be a penis bank in collections.

## Supplementary materials

Supplementary material 1 Gazetteer of collecting localities and specimens examined Data type occurrences, morphological (excel file) Explanation note Species ID, voucher number, diploid number, the origin of specimens (if wild caught or captive born), the crossing origin, if captive-born (generation, parentals, diploid number and locality of parentals), locality with latitude and longitude, the grouping according to the present study. For each specimen, the values of qualitative characters (S = Spines; DC = Dorsal Cleft; DCD = Dorsal Cleft Depth; VC = Ventral Cleft; GS = Glans Shape and DS = Dark Spots), and the mean of quantitative characters, in millimetres (SAL = Spined Area Length; TL = Total Length; BW = Base Width; TW = Tip Width; MW = Middle width; DCL = Dorsal Cleft Length; MBMW = Medial Bacular Mound Width Wild. https://binary.pensoft.net/file/777797This dataset is made available under the Open Database License (http://opendatacommons.org/licenses/odbl/1.0/). The Open Database License (ODbL) is a license agreement intended to allow users to freely share, modify, and use this Dataset while maintaining this same freedom for others, provided that the original source and author(s) are credited.Leonardo Campana, Letícia Rosário Cruz, Roberta Paresque, Valéria Fagundes

Supplementary material 2 Frequency of characters Data type table (word document) Explanation note Frequency of the presence of the characters states in *A.cursor*, *A.montensis* and hybrids. https://binary.pensoft.net/file/777798This dataset is made available under the Open Database License (http://opendatacommons.org/licenses/odbl/1.0/). The Open Database License (ODbL) is a license agreement intended to allow users to freely share, modify, and use this Dataset while maintaining this same freedom for others, provided that the original source and author(s) are credited.Leonardo Campana, Letícia Rosário Cruz, Roberta Paresque, Valéria Fagundes

Supplementary material 3 Comparing data from *A.cursor* individuals from Pernambuco (ACU^PE^) and *A.montensis* from São Paulo (AMO^SP^) Data type table (word document) Explanation note Independent samples t-test between wild-type group and captive-born group. The upper table compared *A.cursor* individuals from Pernambuco (ACU^PE^) and the lower table compared *A.montensis* individuals from São Paulo (AMO^SP^). https://binary.pensoft.net/file/777799This dataset is made available under the Open Database License (http://opendatacommons.org/licenses/odbl/1.0/). The Open Database License (ODbL) is a license agreement intended to allow users to freely share, modify, and use this Dataset while maintaining this same freedom for others, provided that the original source and author(s) are credited.Leonardo Campana, Letícia Rosário Cruz, Roberta Paresque, Valéria Fagundes

Supplementary material 4 Photographs of glans penis of both species and hybrids Data type Morphological images (PDF file) Explanation note Photographs of glans penis using the extended focus imaging system GT-Vision (Leica microsystems). PE = Pernambuco; ES = Espírito Santo; BA = Bahia; SP = São Paulo and MG = Minas Gerais. *Akodoncursor*, cylindric shape. 1. Dorsal and 2. Ventral views, LGA 5064, ES. 3. Dorsal and 4. Ventral views, LGA 5052, PE. 5. Dorsal and 6. Ventral views, LGA 4645, BA. 7. Dorsal view, LGA 4987, PE. 8. Dorsal view, LGA 4968, ES. 9. Dorsal view, LGA 5113, PE. 10. Dorsal view, LGA 5111, PE. 11. Dorsal view, LGA 5106, ES. 12. Dorsal view, LGA 5107, ES. 13. Dorsal view, LGA 4083, BA. 14. Dorsal view, LGA 4216, BA. 15. Dorsal view, LGA 4282, BA. 16. Dorsal view, LGA 4471, ES. 17. Dorsal view, LGA 5110, PE. *Akodonmontensis*, barrel shape. 18. Dorsal and 19. Lateral views, LGA 5245, SP. 20. Dorsal view, LGA 4878, SP. 21. Dorsal view, LGA 4870, SP. 20. Dorsal view, LGA 4869, SP. 23. Dorsal view, LGA 4867, SP. 20. Dorsal view, LGA 4878, SP. 24. Dorsal view, LGA 5186, SP×MG. 25. Dorsal view, LGA 4866, SP×MG. Ventral view. *A.montensis* and presence of dark spots. 26. LGA 5245, SP. 27. LGA 4810, SP. 28. LGA 4867, SP. 29. LGA 4803, SP. 30. LGA 4869, SP. 31. 4870, SP. Ventral view. *A.cursor* and lack of dark spots. 32. LGA 5120, PE. 33. LGA 5025, PE. 34. LGA 4645, BA. 35. LGA 5064, ES. Hybrids, dorsal, lateral and ventral views, respectively. 36–38. LGA 4403, hybrid ACU×AMO. 39–41. LGA 5154, hybrid AMO×ACU. Hybrids, dorsal and ventral views, respectively. 42–43. LGA 5155, hybrid AMO×ACU. https://binary.pensoft.net/file/777800This dataset is made available under the Open Database License (http://opendatacommons.org/licenses/odbl/1.0/). The Open Database License (ODbL) is a license agreement intended to allow users to freely share, modify, and use this Dataset while maintaining this same freedom for others, provided that the original source and author(s) are credited.Leonardo Campana, Letícia Rosário Cruz, Roberta Paresque, Valéria Fagundes

## References

[B1] Abreu MSL Machado R Barbieri F Freitas NS Oliveira LR (2013) Anomalous colour in Neotropical mammals: a review with new records for *Didelphis* sp. (Didelphidae: Didelphimorphia) and *Arctocephalusaustralis* (Otariidae: Carnivora). Brazilian Journal of Biology 73(1): 185–194. 10.1590/S1519-69842013000100020 23644801

[B2] Adebayo AO Akinloye AK Olurode SA Anis EO Oke BO (2011) The structure of the penis with the associated baculum in the male greater cane rat (*Thryonomysswinderianus*). Folia Morphologica 70(3): 197–203. 21866532

[B3] Altuna CA Lessa EP (1985) Penial morphology in Uruguayan species of *Ctenomys* (Rodentia: Octodontidae). Journal of Mammalogy 66(3): 483–488. 10.2307/1380923

[B4] Astúa D Bandeira I Geise L (2015) Cranial morphometric analyses of the cryptic rodent species *Akodoncursor* and *Akodonmontensis* (Rodentia, Sigmodontinae). Oecologia Australis 19(1): 143–157. 10.4257/oeco.2015.1901.09

[B5] Auffray JC Alibert P Latieule C Dod B (1996) Relative warp analysis of skull shape across the hybrid zone of the house mouse (*Musmusculus*) in Denmark. Journal of Zoology 240(3): 441–455. 10.1111/j.1469-7998.1996.tb05297.x

[B6] Bachanek J Postawa T (2010) Morphological evidence for hybridization in the sister species *Myotismyotis* and *Myotisoxygnathus* (Chiroptera: Vespertilionidae) in the Carpathian Basin. Acta Chiroptogica 12(2): 439–448. 10.3161/150811010X538007

[B7] Balbontin J Reig S Moreno S (1996) Evolutionary relationships of *Ctenomys* (Rodentia: Octodontidae) from Argentina, based on penis morphology. Acta Theriologica 41: 237–253. 10.4098/AT.arch.96-25

[B8] Brandão MV Percequillo AR D’Elía G Paresque R Carmignotto AP (2021) A new species of *Akodon* Meyen, 1833 (Rodentia: Cricetidae: Sigmodontinae) endemic from the Brazilian Cerrado. Journal of Mammalogy 102(1): 101–122. 10.1093/jmammal/gyaa126

[B9] Brandão MV Carmignotto AP Percequillo AR Christoff AU Mendes-Oliveira AC Geise L (2022) A new species of *Akodon* Meyen, 1833 (Rodentia: Cricetidae) from dry forests of the Amazonia-Cerrado transition. Zootaxa 5205(5): 401–435. 10.11646/zootaxa.5205.5.1 37045425

[B10] Colombi VH Lopes SR Fagundes V (2010) Testing the Rio Doce as a riverine barrier in shaping the Atlantic Rainforest population divergence in the rodent *Akodoncursor*. Genetics and Molecular Biology 33(4): 785–789. 10.1590/S1415-47572010000400029 21637592 PMC3036154

[B11] Comelis MT Bueno LM Góes RM Taboga SR Morielle-Versute E (2018) Morphological and histological characters of penile organisation in eleven species of molossid bats. Zoology (Jena, Germany) 127: 70–83. 10.1016/j.zool.2018.01.006 29500059

[B12] Cserkész T Rusin M Sramkó G (2016) An integrative systematic revision of the European southern birch mice (Rodentia: Sminthidae, *Sicistasubtilis* group). Mammal Review 46(2): 114–130. 10.1111/mam.12058

[B13] Cserkész T Fülöp A Almerekova S Kondor T Laczkó L Sramkó G (2019) Phylogenetic and morphological analysis of birch mice (Genus *Sicista*, Family Sminthidae, Rodentia) in the Kazak Cradle with description of a new species. Journal of Mammalian Evolution 26(1): 147–163. 10.1007/s10914-017-9409-6

[B14] Fagundes V Nogueira CDA (2007) The use of PCR-RFLP as an identification tool for three closely related species of rodents of the genus *Akodon* (Sigmodontinae, Akodontini). Genetics and Molecular Biology 30(3): 698–701. 10.1590/S1415-47572007000400031

[B15] Fagundes V Vianna-Morgante AM Yonenaga-Yassuda Y (1997) Telomeric sequences localization and G-banding patterns in the identification of a polymorphic chromosomal rearrangement in the rodent *Akodoncursor* (2n = 14, 15 and 16). Chromosome Research 5(4): 228–232. 10.1023/A:1018463401887 9244449

[B16] Fagundes V Christoff AU Yonenaga-Yassuda Y (1998) Extraordinary chromosomal polymorphism with 28 different karyotypes in the neotropical species *Akodoncursor* (Muridae, Sigmodontinae), one of the smallest diploid numbers in rodents (2n = 16, 15 and 14). Hereditas 129(3): 263–274. 10.1111/j.1601-5223.1998.00263.x 10319722

[B17] Fasel NJ Mamba ML Monadjem A (2020) Penis morphology facilitates identification of cryptic African bat species. Journal of Mammalogy 101(5): 1392–1399. 10.1093/jmammal/gyaa073

[B18] Geise L Weksler M Bonvicino CR (2004) Presence or absence of gallbladder in some Akodontini rodents (Muridae, Sigmodontinae). Mammalian Biology 69(3): 210–214. 10.1078/1616-5047-00136

[B19] Geise L Moraes DA Silva HS (2005) Morphometric differentiation and distributional notes of three species of *Akodon* (Muridae, Sigmodontinae, Akodontini) in the Atlantic coastal area of Brazil. Arquivos do Museu Nacional 63(1): 63–74.

[B20] Gentile R D’Andrea PS Cerqueira R (1997) Home ranges of *Philanderfrenata* and *Akodoncursor* in a Brazilian restinga (coastal shrubland). Mastozoología Neotropical 4: 105–112.

[B21] Gonçalves PR Myers P Vilela JF Oliveira JA (2007) Systematics of species of the genus *Akodon* (Rodentia: Sigmodontinae) in southeastern Brazil and implications for the biogeography of the Campos de Altitude. Miscellaneous Publications, Museum of Zoology. University of Michigan 197: 1–24.

[B22] Hammer Ø Harper DA Ryan PD (2001) PAST: Paleontological statistics software package for education and data analysis. Palaeontologia Electronica 4(1): 9.

[B23] Hooper ET (1958) The male phallus in mice of the genus *Peromyscus*. Miscellaneous Publications, Museum of Zoology, University of Michigan 105: 1–40.

[B24] Hooper ET Musser GG (1964) The glans penis in Neotropical cricetines (Family Muridae) with comments on classification of muroid rodents. Miscellaneous Publications, Museum of Zoology, University of Michigan 123: 1–57.

[B25] IBM (2019) IBM SPSS statistics. Ver. 26.0. IBM Corporation, Armonk, NY.

[B26] Kubiak BB Kretschmer R Leipnitz LT Maestri R de Almeida TS Borges LR de Freitas TRO (2020) Hybridization between subterranean tuco-tucos (Rodentia, Ctenomyidae) with contrasting phylogenetic positions. Scientific Reports 10(1): 1–13. 10.1038/s41598-020-58433-5 32001746 PMC6992752

[B27] Lidicker Jr WZ (1968) A phylogeny of New Guinea rodent genera based on phallic morphology. Journal of Mammalogy 49(4): 609–643. 10.2307/1378724

[B28] Maestri R Fornel R Gonçalves GL Geise L de Freitas TRO Carnaval AC (2016) Predictors of intraspecific morphological variability in a tropical hotspot: Comparing the influence of random and non random factors. Journal of Biogeography 43(11): 2160–2172. 10.1111/jbi.12815

[B29] Parag A Bennett NC Faulkes CG Bateman PW (2006) Penile morphology of African mole rats (Bathyergidae): Structural modification in relation to mode of ovulation and degree of sociality. Journal of Zoology 270(2): 323–329. 10.1111/j.1469-7998.2006.00141.x

[B30] Pardiñas UF Teta P Alvarado-Serrano D Geise L Jayat JP Ortiz PE Gonçalves PR D’Elía G (2015) Genus *Akodon* Meyen. In: Patton JL Pardiñas UF D’Elía G (Eds) Mammals of South America, Volume 2: Rodents.University of Chicago Press, Chicago, 144–204.

[B31] Renaud S Alibert P Auffray JC (2012) Modularity as a source of new morphological variation in the mandible of hybrid mice. BMC Evolutionary Biology 12(1): 141. 10.1186/1471-2148-12-141 22873779 PMC3506452

[B32] Rocha-Barbosa O Bernardo JL Loguercio MFC Freitas TRO Santos-Mallet JR Bidau CJ (2013) Penial morphology in three species of Brazilian Tuco-tucos, *Ctenomystorquatus*, *C.minutus*, and *C.flamarioni* (Rodentia: Ctenomyidae). Brazilian Journal of Biology 73(1): 201–209. 10.1590/S1519-69842013000100022 23644803

[B33] Rohlf FJ (2009) TPSDig. Ver. 2.14. State University of New York, Stony Brook, NY.

[B34] Rosalino LM Martin PS Gheler-Costa C Lopes PC Verdade LM (2013) Allometric relations of Neotropical small rodents (Sigmodontinae) in anthropogenic environments. Zoological Science 30(7): 585–590. 10.2108/zsj.30.585 23829219

[B35] Sbalqueiro IJ Nascimento AP (1996) Occurrence of *Akodoncursor* (Rodentia, Cricetidae) with 14, 15 and 16 chromosome cytotypes in the same geographic area in southern Brazil. Revista Brasileira de Genetica 19(4): 565–569. 10.1590/S0100-84551996000400005

[B36] Silva FA Lessa G Bertuol F Freitas TRO Quintela FM (2020) Chromatic anomalies in Akodontini (Cricetidae: Sigmodontinae). Brazilian Journal of Biology 80(2): 479–481. 10.1590/1519-6984.214680 31291406

[B37] Silveira F Sbalqueiro IJ Monteiro-Filho ELDA (2013) Identification of the Brazilian species of *Akodon* (Rodentia: Cricetidae: Sigmodontinae) through the microstructure of the hair. Biota Neotropica 13(1): 339–345. 10.1590/S1676-06032013000100033

[B38] Soares AA Castro JP Balieiro P Dornelles S Degrandi TM Sbalqueiro IJ Hass I (2018) B Chromosome diversity and repetitive sequence distribution in an isolated population of *Akodonmontensis* (Rodentia, Sigmodontinae). Cytogenetic and Genome Research 154(2): 79–85. 10.1159/000487471 29544219

[B39] Valdez L D’Elía G (2013) Differentiation in the Atlantic Forest: Phylogeography of *Akodonmontensis* (Rodentia, Sigmodontinae) and the Carnaval–Moritz model of Pleistocene refugia. Journal of Mammalogy 94(4): 911–922. 10.1644/12-MAMM-A-227.1

