# Supplementary material for: ﻿Penile shape discriminates two cryptic species of Akodon Meyen, 1833 (Mammalia, Rodentia, Cricetidae) from eastern Brazil
Source: Zookeys. 2022 Dec 5;1134:1–22. doi: 10.3897/zookeys.1134.89587 (PMC9836724; doi:10.3897/zookeys.1134.89587)
Supplement: Supplementary material 2 — Frequency of characters [file zookeys-1134-001_article-89587__-s002.docx]

**SUPPLEMENTARY DATA 2**

Independent samples t-test between wild-type group and captive-born group. The first table is comparing *A. cursor* wild-type from Pernambuco individuals (ACU^PE^w) and *A. cursor* captive-born individuals generated by experimental crossings between individuals from Pernambuco (ACU^PE^c). The second Table is comparing *A. montensis* wild-type from São Paulo individuals (AMO^SP^wt) and *A. montensis* captive-born individuals generated by experimental crossings between individuals from São Paulo (AMO^SP^cb).

|  | **ACU^PE^w**  **(n=9)** | **ACU^PE^c**  **(n=12)** | **t-test** | | |
| --- | --- | --- | --- | --- | --- |
|  |  |  | **t** | **d.f.** | **p** |
| SAL | 4.91044 (±0.26732) | 4.80817 (±0.42317) | -0.634 | 19 | 0.534 |
| TL | 5.52578 (±0.26683) | 5.61400 (±0.26711) | 0.749 | 19 | 0.463 |
| BW | 2.65756 (±0.19331) | 2.48667 (±0.27080) | -1.607 | 19 | 0.125 |
| TW | 1.82867 (±0.30817) | 1.96867 (±0.27682) | 1.093 | 19 | 0.288 |
| MW | 3.06689 (±0.16080) | 2.96100 (±0.41871) | -0.801 | 14 | 0.436 |
| DCL | 2.28525 (±0.19982) | 2.61300 (±0.38009) | 2.159 | 14 | 0.049 |
| MBMW | 0.64725 (±0.09213) | 0.62333 (±0.10825) | -0.512 | 18 | 0.615 |
|  | **AMO^SP^w**  **(n=3)** | **AMO^SP^c**  **(n=14)** | **t-test** | | |
|  |  |  | **t** | **d.f** | **p** |
| SAL | 5.04800 (±0.35261) | 4.74500 (±0.33160) | 1.424 | 15 | 0.175 |
| TL | 5.16000 (±0.38025) | 5.30743 (±0.24427) | -0.870 | 15 | 0.398 |
| BW | 2.87933 (±0.11906) | 2.79443 (±0.21912) | 0.640 | 15 | 0.532 |
| TW | 1.82800 (±0.11886) | 2.05857 (±0.40478) | -0.955 | 15 | 0.355 |
| MW | 3.19000 (±0.29813) | 3.16386 (±0.30726) | 0.134 | 15 | 0.895 |
| DCL | 2.61667 (±0.28999) | 2.76691 (±0.52435) | -0.468 | 12 | 0.648 |
| MBMW | 0.46400 (±0.31678) | 0.66071 (±0.14667) | -1.580 | 14 | 0.137 |

Groups Abbreviation: (ACU^PE^w) *A. cursor* wild-type individuals from Pernambuco; (ACU^PE^c) *A. cursor* captive-born individuals with parentals from Pernambuco; (AMO^SP^w) *A. montensis* wild-type individuals from São Paulo; (AMO^SP^c) *A. montensis* captive-born individuals with parentals from São Paulo. Characters Abbreviation: (SAL) Spined Area Length; (TL) Total Length; (BW) Base Width; (TW) Tip Width; (MW) Middle width; (DCL) Dorsal Cleft Length; (MBMW) Medial Bacular Mound Width. Values are Means (±Standard Deviation).
