# Supplementary material for: ﻿Penile shape discriminates two cryptic species of Akodon Meyen, 1833 (Mammalia, Rodentia, Cricetidae) from eastern Brazil
Source: Zookeys. 2022 Dec 5;1134:1–22. doi: 10.3897/zookeys.1134.89587 (PMC9836724; doi:10.3897/zookeys.1134.89587)
Supplement: Supplementary material 3 — Comparing data from A.cursor individuals from Pernambuco (ACUPE) and A.montensis from São Paulo (AMOSP) [file zookeys-1134-001_article-89587__-s003.docx]

**SUPPLEMENTARY DATA 3**

Table S3 Frequency of the presence of the characters states in *A. cursor*, *A. montensis* and hybrids.

| **Group** | **subgroup^#^** | **sample** | **Character states*** | **S** | **DC** | **DCD** | **VC** | **GS** | **DS** |
| --- | --- | --- | --- | --- | --- | --- | --- | --- | --- |
| *A. cursor* | ACU^BA^ | 24 | 0 | 65.2 % | 0.0 % | 26.0 % | 72.2 % | 100.0 % | 100.0 % |
|  |  |  | 1 | 30.4 % | 26.0 % | 60.8 % | 22.2 % | 0.0 % | 0.0 % |
|  |  |  | 2 | 4.3 % | 73.9 % | 13.0 % | 5.5 % | - | - |
|  | ACU^PE^ | 21 | 0 | 45.4 % | 0.0 % | 18.1 % | 50.0 % | 100.0 % | 100.0 % |
|  |  |  | 1 | 54.5 % | 9.0 % | 45.4 % | 40.0 % | 0.0 % | 0.0 % |
|  |  |  | 2 | 0.0 % | 90.9 % | 36.3 % | 10.0 % | - | - |
|  | ACU^ES^ | 11 | 0 | 55.0 % | 10.5 % | 33.3 % | 62.5 % | 100.0 % | 100 % |
|  |  |  | 1 | 40.0 % | 42.1 % | 33.3 % | 37.5 % | 0.0 % | 0.0 % |
|  |  |  | 2 | 5.0 % | 47.3 % | 33.3 % | 0.0 % | - | - |
| *A. montensis* | AMO^SP^ | 16 | 0 | 15.4 % | 8.3 % | 0.0 % | 27.2 % | 0.0 % | 27.2 % |
|  |  |  | 1 | 15.4 % | 33.3 % | 33.3 % | 9.0 % | 100.0 % | 72.7 % |
|  |  |  | 2 | 69.2 % | 58.3 % | 66.6 % | 63.6 % | - | - |
|  | AMO^SPxMG^ | 11 | 0 | 10.0 % | 0.0 % | 0.0 % | 27.2 % | 0.0 % | 36.3 % |
|  |  |  | 1 | 10.0 % | 11.1 % | 33.3 % | 27.2 % | 100.0 % | 63.6 % |
|  |  |  | 2 | 80.0 % | 88.8 % | 66.6 % | 45.4 % | - | - |
| Hybrids | HYB^ACUxAMO^ | 7 | 0 | 0.0 % | 0.0% | 0.0 % | 43.0 % | 29.0% | 71.0 % |
|  |  |  | 1 | 29.0 % | 29.0 % | 43.0 % | 43.0 % | 71.0 % | 29.0 % |
|  |  |  | 2 | 71.0 % | 71.0% | 57.0 % | 14.0 % | - | - |
|  | HYB^AMOxACU^ | 6 | 0 | 0.0 % | 0.0 % | 0.0 % | 17.0 % | 50.0 % | 100.0 % |
|  |  |  | 1 | 67.0 % | 17.0 % | 17.0 % | 50.0 % | 50.0 % | 0.0 % |
|  |  |  | 2 | 33.0 % | 83.0 % | 83.0 % | 33.0 % | - | - |

#As Table 1. *As Table 2. Characters Abbreviation: S = Spines; DC = Dorsal Cleft; DCD = Dorsal Cleft Depth; VC = Ventral Cleft; GS = Glans Shape and DS = Dark Spots. *A. cursor* groups: Individuals from Pernambuco (ACU^PE^); individuals from Bahia (ACU^BA^) and individuals from Espírito Santo (ACU^ES^). *A. montensis* individuals from São Paulo (AMO^SP^) and individuals from crossings between individuals from São Paulo and Minas Gerais (AMO^SPxMG^).
