# Supplementary material for: ﻿Penile shape discriminates two cryptic species of Akodon Meyen, 1833 (Mammalia, Rodentia, Cricetidae) from eastern Brazil
Source: Zookeys. 2022 Dec 5;1134:1–22. doi: 10.3897/zookeys.1134.89587 (PMC9836724; doi:10.3897/zookeys.1134.89587)
Supplement: Supplementary material 4 — Photographs of glans penis of both species and hybrids [file zookeys-1134-001_article-89587__-s004.pdf]

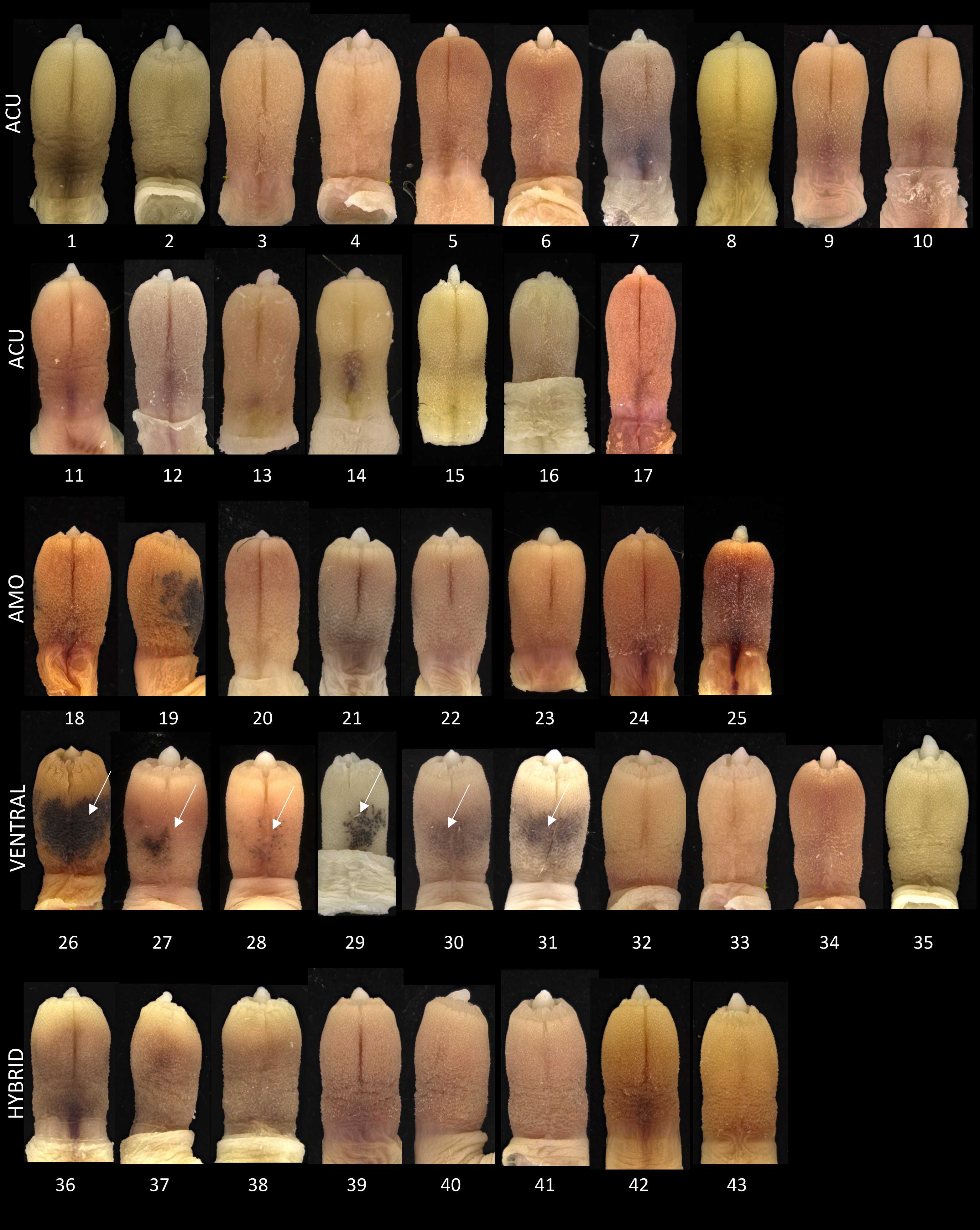

ACU
1
2
3
4
5
6
7
8
9
10
ACU
11
12
13
14
15
16
17
AMO
18
19
20
21
22
23
24
25
VENTRAL
26
27
28
29
30
31
32
33
34
35
HYBRID
36
37
38
39
40
41
42
43

Supplementary Material S4. Photographs of glans penis using the extended focus imaging system GT-Vision (©Leica microsystems). PE = Pernambuco; ES = Espírito santo; BA = Bahia; SP = São Paulo and MG = Minas Gerais.

***Akodon cursor*, cylindric shape**. 1. Dorsal and 2. Ventral views, LGA 5064, ES. 3. Dorsal and 4. Ventral views, LGA 5052, PE. 5. Dorsal and 6. Ventral views, LGA 4645, BA. 7. Dorsal view, LGA 4987, PE. 8. Dorsal view, LGA 4968, ES. 9. Dorsal view, LGA 5113, PE. 10. Dorsal view, LGA 5111, PE. 11. Dorsal view, LGA 5106, ES. 12. Dorsal view, LGA 5107, ES. 13. Dorsal view, LGA 4083, BA. 14. Dorsal view, LGA 4216, BA. 15. Dorsal view, LGA 4282, BA. 16. Dorsal view, LGA 4471, ES. 17. Dorsal view, LGA 5110, PE.
***Akodon montensis*, barrel shape**. 18. Dorsal and 19. Lateral views, LGA 5245, SP. 20. Dorsal view, LGA 4878, SP. 21. Dorsal view, LGA 4870, SP. 20. Dorsal view, LGA 4869, SP. 23. Dorsal view, LGA 4867, SP. 20. Dorsal view, LGA 4878, SP. 24. Dorsal view, LGA 5186, SPxMG. 25. Dorsal view, LGA 4866, SPxMG.
Ventral view. *A. montensis* and presence of dark spots. 26. LGA 5245, SP. 27. LGA 4810, SP. 28. LGA 4867, SP. 29. LGA 4803, SP. 30. LGA 4869, SP. 31. 4870, SP.
Ventral view. *A. cursor* and lack of dark spots. 32. LGA 5120, PE. 33. LGA 5025, PE. 34. LGA 4645, BA. 35. LGA 5064, ES.
Hybrids, dorsal, lateral and ventral views, respectively. 36-38. LGA 4403, hybrid ACUxAMO. 39-41. LGA 5154, hybrid AMOxACU.
Hybrids, dorsal and ventral views, respectively. 42-43. LGA 5155, hybrid AMOxACU.
